# Polymer–Surfactant System Based Amorphous Solid Dispersion: Precipitation Inhibition and Bioavailability Enhancement of Itraconazole

**DOI:** 10.3390/pharmaceutics10020053

**Published:** 2018-04-24

**Authors:** Disang Feng, Tingting Peng, Zhengwei Huang, Vikramjeet Singh, Yin Shi, Ting Wen, Ming Lu, Guilan Quan, Xin Pan, Chuanbin Wu

**Affiliations:** School of Pharmaceutical Sciences, Sun Yat-sen University, Guangzhou 510006, China; disyfeng@gmail.com (D.F.); pengtt6@mail.sysu.edu.cn (T.P.); hzhengw3@mail2.sysu.edu.cn (Z.H.); kasana.chem@gmail.com (V.S.); shiy53@mail2.sysu.edu.cn (Y.S.); went5@mail2.sysu.edu.cn (T.W.); luming3@mail.sysu.edu.cn (M.L.); wuchuanb@mail.sysu.edu.cn (C.W.)

**Keywords:** amorphous solid dispersion, precipitation, TPGS, HPMC AS, itraconazole, bioavailability

## Abstract

The rapid release of poorly water-soluble drugs from amorphous solid dispersion (ASD) is often associated with the generation of supersaturated solution, which provides a strong driving force for precipitation and results in reduced absorption. Precipitation inhibitors, such as polymers and surfactants, are usually used to stabilize the supersaturated solution by blocking the way of kinetic or thermodynamic crystal growth. To evaluate the combined effect of polymers and surfactants on maintaining the supersaturated state of itraconazole (ITZ), various surfactants were integrated with enteric polymer hydroxypropyl methylcellulose acetate succinate (HPMC AS) to develop polymer–surfactant based solid dispersion. The supersaturation stability was investigated by in vitro supersaturation dissolution test and nucleation induction time measurement. Compared to the ASD prepared with HPMC AS alone, the addition of d-alpha-tocopheryl polyethylene glycol 1000 succinate (TPGS) exhibited a synergistic effect on precipitation inhibition. The results indicated that the TPGS not only significantly reduced the degree of supersaturation which is the driving force for precipitation, but also provided steric hindrance to delay crystal growth by absorbing onto the surface of small particles. Subsequently, the formulations were evaluated in vivo in beagle dogs. Compared with commercial product Sporanox^®^, the formulation prepared with HPMC AS/TPGS exhibited a 1.8-fold increase in the AUC (0–24 h) of ITZ and a 1.43-fold increase of hydroxyitraconazole (OH-ITZ) in the plasma. Similarly, the extent of absorption was increased by more than 40% when compared to the formulation prepared with HPMC AS alone. The results of this study demonstrated that the ASD based on polymer–surfactant system could obviously inhibit drug precipitation in vitro and in vivo, which provides a new access for the development of ASD for poorly water-soluble drug.

## 1. Introduction

Amorphous solid dispersion (ASD) has attracted considerable attention as a promising strategy to improve the dissolution and bioavailability of poorly water-soluble active pharmaceutical ingredients (APIs) [1,2,3]. The enhanced dissolution can be attributed to the following several perspectives: (1) the increased wettability by surrounding hydrophilic carriers [4], (2) changes in the physical state of the drug from crystalline to amorphous form [2], (3) reduction in drug agglomeration [5]. After oral administration, rapid drug release is induced when the solid dispersion is subjected into the gastrointestinal tract (GI) and a supersaturated solution exceeding the equilibrium solubility will be generated. Theoretically, drugs at high concentration have an increased driving force for flux across the gastrointestinal membrane, and improved oral absorption could be achieved [6]. However, for drugs in the supersaturated state, possessing higher chemical potential tends to precipitate into an energetically more favorable crystalline form, which may jeopardize absorption and result in compromised bioavailability [7,8]. More risk of precipitation may occur for poorly water-soluble weakly base drugs, which have a sharp decreased solubility transition from gastric to intestinal fluid [9,10]. Therefore, it is essential to maintain the supersaturated state for sufficient time period to achieve better absorption.

In recent years, numerous studies have been conducted to inhibit and/or reduce the drug precipitation using various excipients as precipitation inhibitors [9,11,12]. Precipitation inhibition may be realized by blocking the way of kinetic or thermodynamic crystal growth [13]. Polymers, an important component of ASDs, have been widely used as kinetic precipitation inhibitors by increasing the viscosity to decrease the molecular mobility and diffusion coefficient [14]. Moreover, the absorption of polymer onto the surface of crystal can also act as mechanical barrier to inhibit the aggregation of nuclei units [8,15]. Consequently, the rate of nucleation and crystal growth can be reduced. Conversely, thermodynamic inhibition of drug precipitation is achieved via reducing the degree of supersaturation degree (*S*) [16]. *S* is one of the most important driving forces for precipitation which can be expressed as

(1) $$S=C/C_{S}$$

where *C* and *C_S_* represent the total drug concentration and the saturation drug concentration, respectively. Therefore, solubilization agents such as surfactants can be used as thermodynamic inhibitors to increase saturation solubility and subsequently reduce the degree of supersaturation. Li et al. reported that Pluronic F127 and TPGS could maintain the supersaturated state for more than 2 h, and the pharmacokinetic study showed that the bioavailability of the formulation containing surfactant was two-fold greater than that of suspension control [17].

Recently, the majority of the published literatures or marketed ASDs have been focused on the binary or ternary drug-polymer system to inhibit the drug precipitation in supersaturated solution [15]. However, it is inadvisable to neglect the role of solubilization agents since the predominance of thermodynamic or kinetic factor was unknown in prohibiting the nucleation growth. Several previous studies have clearly demonstrated that surfactants could effectively stabilize the supersaturated state of poorly water-soluble drugs, such as dutasteride [18], celecoxib [19], and tacrolimus [20]. Hence, more attention should be paid to the effect of surfactants on nucleation and crystal growth.

Previous studies have demonstrated that the combination of polymer and surfactant could prolong drug precipitation induction time and resulted in higher bioavailability [21]. However, the surfactants were pre-dissolved in the dissolution test medium instead of directly formulating with polymers and drugs [21,22]. It is unfeasible for such a dosage form to be formulated as a capsule or tablet. To create a feasible polymer–surfactant system, polymer, surfactant and drug were directly formulated as ASD using hot melt extrusion in this study.

Therefore, the objective of this study was to establish a polymer–surfactant system based on ASD to improve the dissolution and bioavailability of poorly water-soluble drugs. Itraconazole (ITZ) was used as a poorly water-soluble model drug. Due to its weakly basic nature with two nitrogen atoms (pKa 2 and 3.7) that can be protonated in the physiological range, ITZ exhibits a pH-dependent solubility. ITZ contained ASD could dissolve in gastric environment to generate a supersaturated solution. However, rapid precipitation was observed when it was subjected to the intestine due to a sharp pH change, leading to low oral bioavailability. The in vitro supersaturation dissolution and nucleation induction time measurement were conducted to investigate the effect of polymer–surfactant complex on the drug precipitation. Furthermore, the oral bioavailability of the optimized formulation was compared with the formulation without surfactant and commercial product Sporanox^®^ in beagle dogs to establish the in vitro–in vivo correlation.

## 2. Materials and Methods

### 2.1. Materials

ITZ of pharmaceutical grade was purchased from Sanlian Pharmaceutical (Harbin, China), its active metabolite hydroxyitraconazole (OH-ITZ) (purity higher than 99%) was purchased from J&K Scientific Ltd. (Shanghai, China) and used as a reference substance. Commercial Sporanox^®^ capsules were obtained from Xian Janssen Pharmaceutical Ltd. (Xian, China). d-α-tocopheryl polyethylene glycol 1000 succinate (TPGS), Poloxamer 188, and sodium dodecyl sulfate (SDS) were purchased from BASF (Ludwigshafen, Germany), Macklin Biochemical Ltd. (Shanghai, China), and FuchenChemical Reagents Factory (Tianjin, China), respectively. Hydroxypropylmethylcellulose acetate succinate (HPMC AS) (grade LF) was kindly provided by Shin Etsu (Shin-Etsu Chemical Co., Ltd., Tokyo, Japan). The beagle dogs used for in vivo bioavailability study were purchased from the ChaiMen Biological Technology Ltd. (Nanjing, China). HCl was obtained from Jinkun chemical co., Ltd (Bengbu, China), the Na_3_PO_4_, NaH_2_PO_4_ and Na_2_HPO_4_ were purchased from Damao Chemical Reagent Factory (Tianjin, China). All reagents were of grade analytical and used without further purification.

### 2.2. Equilibrium Solubility

**Figure 1:**
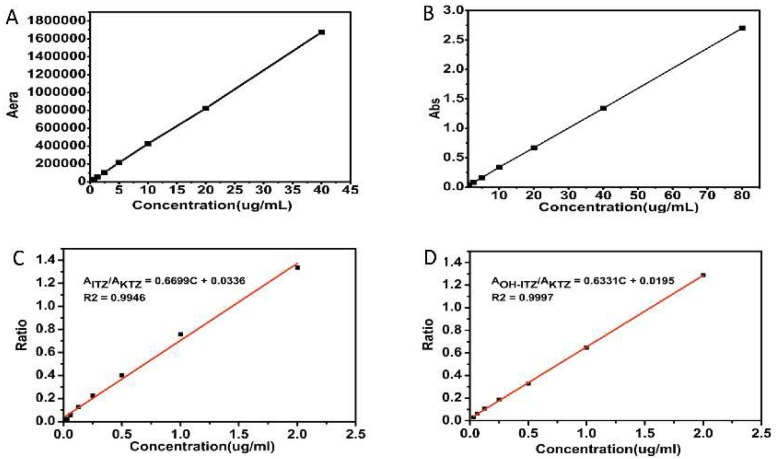
Calibration curves of (**A**) ITZ (itraconazole) in HPLC (high performance liquid), (**B**) ITZ in UV (ultraviolet-visible) spectrophotometry, (**C**) ITZ (plasma) in HPLC, (**D**) OH-ITZ (hydroxyitraconazole) (plasma) in HPLC.

The equilibrium solubility of ITZ in simulated intestinal fluid (SIF, pH 6.8, prepared with 0.1 N HCl and 0.2 M Na_3_PO_4_ at a volume ratio of 3:1) with different kinds of surfactants was determined by the shaking flask method. An excess amount of ITZ was added to SIF with or without pre-dissolved surfactant (1 mg/mL) and mixed by vortex for 2 min. After incubation in a shaking incubator at 37 °C for 48 h, the suspension was centrifuged (15,000 rpm for 10 min) and filtered through a 0.22 μm Millipore filter (Jinteng Experimental Equipment Co., Ltd, Tianjin, China). Prior to assay by high performance liquid (HPLC) (Shimadzu Corporation, Kyoto, Japan) using a PhenomenexC18 column (4.6 × 250 mm, 5 μm), the filtrate was diluted 1:1 with mobile phase. The mobile phase was consisted of 0.5% diisopropylamine in methanol and 0.1% ammonium acetate in distilled water (80:20) with a rate of 1.0 mL/min. The effluent was monitored at an ultraviolet-visible (UV) absorption wavelength of 263 nm. The retention time of ITZ under these conditions was 4.9 min. Calibration curve (Figure 1A) were linear over a concentration range of 0.04–40 μg/mL. The limit of quantitation (LOQ) and limit of detection (LOD) was 25 ng and 10 ng, respectively. Each equilibrium solubility test was performed in triplicate.

### 2.3. Preparation of Solid Dispersions

ASDs were prepared by hot melt extrusion equipped with a conical co-rotating (5/14 mm diameter) twin screw HAAKE MiniLab II Microcompounder (Thermo Electron GmbH, Karlsruhe, Germany). All the formulations were prepared at a fixed drug loading (20%, *w*/*w*) with various ratios of HPMC AS to surfactant (Table 1). Briefly, the physical mixtures prepared by homogenously mixing ITZ and carriers were manually fed into the melt extruder, and the extrusion temperature and screw speed were adjusted to 170 °C and 100 rpm, respectively. The obtained hot melt extrudates were collected and cooled in ambient conditions, milled using a coffee grinder, and passed through a 100-mesh sieve for further analysis. 

### 2.4. Supersaturation Dissolution Test with pH-Shift

In order to simulate the drug peristalsis in vivo situation, a transfer of simulated gastric fluid (SGF) to SIF was conducted for in vitro dissolution experiment. Supersaturation dissolution tests were performed according to USP 29 Apparatus 2 guidelines (paddle method) with a ZRS-8G dissolution tester (TDTF, Tianjin, China). Briefly, samples containing equivalent amounts of ITZ (45 mg) were subjected to each dissolution vessel containing SGF (750 mL of 0.1 N HCl) for 2 h, followed by pH adjustment to 6.8 by the addition of 250 mL of 0.2 M tribasic sodium phosphate (Na_3_PO_4_) for additional 3 h. The dissolution media was kept at 37.0 ± 0.5 °C and the paddle speed was maintained at 100 rpm throughout the test procedure. Aliquot samples (5 mL) were withdrawn at predetermined time intervals (120, 125, 150, 180, 240, and 300 min). The samples were filtered through a 0.22 m millipore filter and the first few drops were discarded. The filtrate was immediately diluted with methanol to prevent the precipitation. The ITZ concentration was assayed by ultraviolet spectrophotometry (TU-1901, Beijing Purkinje General Instrument Co., Ltd., Beijing, China) at a wavelength of 263 nm. The calibration curve of ITZ in UV spectrophotometry was shown in Figure 1B with a concentration range of 0.25–80 μg/mL. The dissolution performance of the marketed product Sporanox^®^, which is a capsule-based formulation containing ASD prepared with HPMC, was also examined under the identical condition. Each dissolution test was performed in triplicate.

### 2.5. Nucleation Induction Time Measurement

Supersaturation stability is commonly evaluated by measuring the induction time for nucleation, which is defined as the time lag for the appearance of the observable crystals [23]. In this study, the precipitation induction time of different formulations were obtained by determining the changes in turbidity using plate reader detection technique. Supersaturated ITZ solutions were prepared by the solvent shift method. All the samples were dissolved in dimethyl sulfoxide (DMSO) to obtain ITZ stock solution at a concentration of 1mg/mL, then the stock solution was added into SIF and stirred constantly to generate a supersaturated solution. Immediately, 300 μL of individual supersaturated solution was transferred into a 96-well polypropylene plate and the optical density value of each well was measured at 490 nm using plate reader (Bio-Tek, Winooski, VT, USA). It should be noted that all the ingredients used in this study does not have any absorbance as checked by plate reader at 490 nm. Thus, the turbidity was the only contributor to the changed absorbance. The samples were also shaken before the measurement and each formulation was detected in triplicate. All the analyses and calculations were carried out using Gen 5.0 (Bio-Tek, Winooski, VT, USA).

### 2.6. Characterization of Optimized ASD

#### 2.6.1. Scanning Electron Microscopy (SEM)

The shape and surface morphology of the ITZ powder, HPMC AS, TPGS, physical mixture, and the optimized ASD formulation were evaluated using SEM (JSM-6330F, Tokyo, Japan). All samples were coated with a thin gold-palladium layer using an automatic magnetron sputter coater system. Then the samples were observed at an acceleration voltage of 10.0 kV.

#### 2.6.2. Differential Scanning Calorimetry (DSC)

Thermal characterization of ITZ, HPMC AS, TPGS, physical mixtures, and optimized ASD formulation were carried out by differential scanning calorimetry (NETZSCH STA- 409 thermogravimetric analyzer, NETZSCH Group, Selb, Germany). Briefly, samples (5–10 mg) were placed in open aluminum pans covered with pierced aluminum lids, progressively heated at a rate of 10 °C/min and subjected to a heat–cool–heat cycle. Nitrogen at a flow rate of 40 mL/min was used as purge gas. Indium was used to calibrate the instrument and the data were analyzed by Proteus analysis software.

#### 2.6.3. Powder X-ray Diffraction (PXRD)

The diffraction patterns of the ITZ powder, excipients, physical mixture and optimized ASD formulation were analyzed by powder X-ray diffraction (Bruker/D2 PHASER, Bremen, Germany) using Cu K radiation at 30 mA and 30 kV, with 2θ in the range of 5–40° at a scanning rate of 5°/min.

#### 2.6.4. Fourier Transform Infrared Spectroscopy (FTIR)

A Fourier transform infrared spectroscope (TEN-SOR37, Bruker, Bremen, Germany) was used to examine the molecular interactions between the drug and excipients in solid state. Each sample was mixed with spectroscopy grade KBr at a weight ratio of 1:5 and then compressed into semitransparent pellet under a pressure of 8 tons for 5 min. The spectra of the samples were examined in the range of 400–4000 cm^−1^ with a resolution of 4 cm^−1^. The software Bruker OPUS 6.5 was used for FTIR data analysis.

### 2.7. In Vivo Bioavailability Study

All the procedures and experimental methods carried out in the present study were approved on 19 October 2016 by the Institutional Animal Care and Use Committee of Sun Yat-sen University (Guangzhou, China), the project identification code is 201610000273. Six healthy beagle dogs (8–10 kg each) were fasted for 12 h prior to the experiment but had free access to water. Beagle dogs were randomly divided into three groups and respectively administered orally with hard capsules containing the optimized polymer–surfactant system, ASD without surfactants (F1) and commercial product Sporanox^®^ equivalent to a dose of 100 mg ITZ. A small quantity of water (approximately 20 mL) was immediately given to ensure that the capsule had been swallowed. Food was returned at 6 h post-dosing. Blood samples (5 mL) were collected from the cephalic vein of the hind leg at 0, 0.5, 1, 2, 3, 4, 6, 8, 10, 12, and 24 h post-dosing. In order to assess the individual variance in the pharmacokinetic profile, a crossover design was carried out with a two-week washout period. The plasma was obtained by centrifugation at 5000 rpm for 15 min. Ketoconazole (KTZ) used as the internal standard was added to the plasma, followed by addition of 0.4 N ZnSO4 solution to alkalize the plasma for further extraction. Finally, the ITZ and ITZ-OH were extracted from the plasma by methyl tert-butyl ether.

#### 2.7.1. Quantitative Analysis of ITZ and OH-ITZ in Plasma

To determine the plasma level of ITZ and its active metabolite OH-ITZ at each time point, a Shimadzu LC-20AT HPLC system (Shimadzu Corporation, Kyoto, Japan) was employed. Separation and quantitation were made on a Symmetry^®^ C18, 4.6 × 250 mm, 5 μm column (Phenomenex, America) with a guard column (4.6 × 12.5 mm, 5 m). The treated plasma sample was filtered through a 0.22 μm Millipore filter before analyzed by HPLC. The mobile phase consisted of acetonitrile: phosphate buffer pH 6.8 (62:38, *v*/*v*) was flowed at a rate of 1.0 mL/min, the phosphate buffer was prepared by 0.2 M NaH_2_PO_4_ and 0.2 M Na_2_HPO_4_ at a volume ratio of 49:51. The retention time of ITZ and OH-ITZ was 13.6 min and 6.8 min, respectively. The eluent was monitored at 263 nm and the column working temperature was kept at 35 °C. The calibration curves of ITZ in plasma sample was shown in Figure 1C, and the LOD and LOQ was 12 ng and 32.8 ng, respectively. As shown in Figure 1D, the LOD and LOQ of OH-ITZ in plasma sample was 22 ng and 41.6 ng, respectively.

#### 2.7.2. Pharmacokinetic Data Analysis and Statistical Analysis

Pharmacokinetic data were calculated and analyzed using a non-compartmental model (WinNonlin; professional edition, V. 3.3, California). The peak plasma concentration (C_max_) and the time of peak plasma concentration (T_max_) were directly obtained from the plasma concentration-time profiles. The area under the concentration–time curve (AUC) from zero to infinity was calculated according to the linear trapezoidal rule. 

The statistical analysis of the mean pharmacokinetic parameters was conducted using a one-way ANOVA test (SPSS 13.0). The post hoc comparison of the means of individual groups was conducted using least significant difference test, and the *p* values of <0.05 were considered significant.

## 3. Results

### 3.1. Selection of Surfactants

The effect of various surfactants on the equilibrium solubility of ITZ in the SIF is presented in Table 2. The equilibrium solubility of ITZ was significantly increased when TPGS, SDS, and poloxamer 188 were added. Particularly, the equilibrium solubility of ITZ was increased to 4.83 μg/mL in the presence of TPGS, showing approximately 580-fold higher than that of SIF (~0.008 μg/mL). In contrast, it was not apparent for the Tween 80, sodium cholate, and Soluplus^®^ to improve the equilibrium solubility of ITZ. It is generally recognized that the excellent solubilizing effect of surfactant can improve drug wettability and inhibit drug precipitation in dissolution medium. Therefore, TPGS, SDS, and poloxamer 188 were selected for further study.

### 3.2. In Vitro Supersaturation Dissolution with pH-Shift

**Figure 2:**
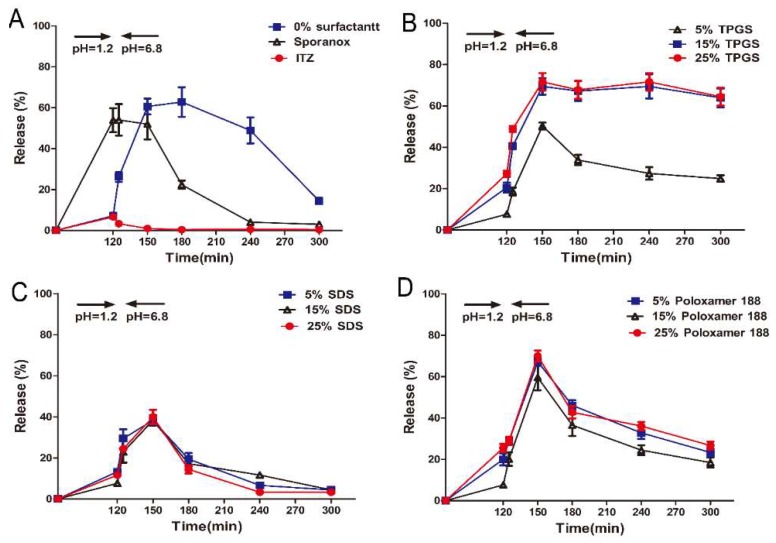
In vitro release profiles of ITZ from different formulations in simulated gastrointestinal fluid (Mean ± S.D., *n* = 3). (**A**) In vitro release profiles of ITZ from pure ITZ, Sporanox^®^, and ASD (amorphous solid dispersion) without surfactant; In vitro release profiles of ITZ from ASD with different amount of (**B**) TPGS (d-α-tocopheryl polyethylene glycol 1000 succinate), (**C**) SDS (sodium dodecyl sulfate), and (**D**) Poloxamer 188.

In the supersaturation dissolution test, samples were agitated in SGF for 120 min, after which the samples were transferred into SIF. As depicted in Figure 2A, approximately 55% of ITZ was released from the marketed product Sporanox^®^ after 2 h in SGF, whereas the release amount from the formulation prepared with HPMC AS (F1) and the crystalline ITZ was only 5% and 3%, respectively. Five minutes after the pH change to 6.8, rapid precipitation was observed in Sporanox^®^ and crystalline ITZ, and the cumulative release amount eventually dropped to less than 3% and 1%, respectively. In contrast, after 30 min switching from SGF to SIF, the cumulative release amount from F1 reached 60%.

In order to investigate the performance of surfactants in inhibiting precipitation, the ASDs were prepared with TPGS, SDS, and poloxamer 188 in different ratio. As shown in Figure 2B, it can be observed that the TPGS formulations (F2, F3 and F4, which was prepared with 5%, 15% and 25% of TPGS, respectively) exhibited the most rapid dissolution in SIF. In particular, approximately 75% of ITZ was released from F3 and F4 at 30 min and the supersaturated state was maintained throughout the whole period of 3 h. In the case of F2, the dissolution of ITZ was rapidly decreased after 30 min in SIF. On the contrary, only 40% and 65% of ITZ was released from the formulations containing SDS (F5–F7) and poloxamer 188 (F8–F10) after 30 min in SIF, respectively (Figure 2C,D), and the release amount drastically reduced to 8% (F5–F7) and 18% (F8–F10).

### 3.3. Nucleation Induction Time Measurement

**Figure 3:**
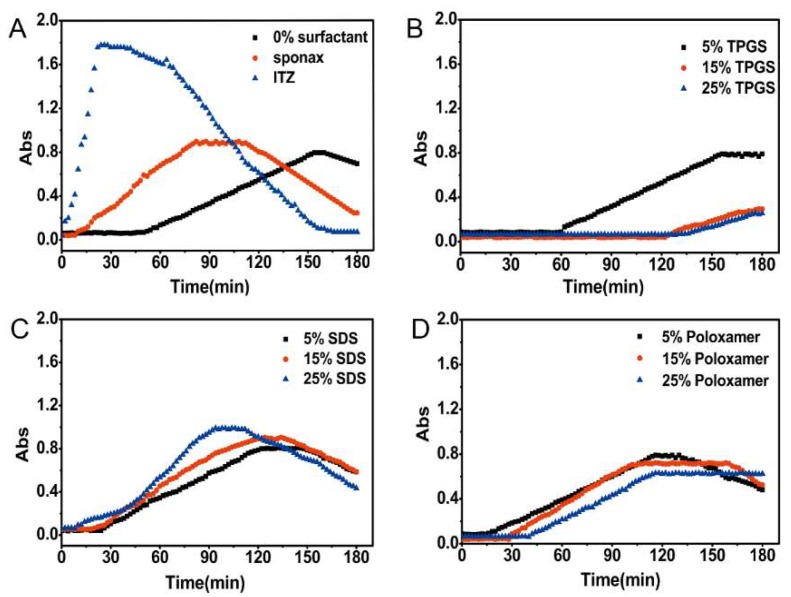
Nucleation induction time for pure ITZ, Sporanox^®^, and different formulations in simulated intestinal fluid. (**A**) Nucleation induction time for pure ITZ (itraconazole), Sporanox^®^, and ASD (amorphous solid dispersion) without surfactant; Nucleation induction time for ASD with different amount of (**B**) TPGS (d-α-tocopheryl polyethylene glycol 1000 succinate), (**C**) SDS (sodium dodecyl sulfate), and (**D**) Poloxamer 188.

The nucleation induction time was measured to investigate the impact of surfactants on the precipitation tendency. The absorbance-time curves of different formulations were shown in Figure 3. After the addition of the stock solution to SIF, the initial solution was transparent and the intensity of absorbance was close to zero. The pure ITZ and commercial product Sporanox^®^ showed rapid increase in the absorbance followed by a gradual decline. The absorbance of the formulation without surfactant (F1) also started to slightly increase at 36 min. It is reasonably indicated that after the addition of the stock solution to SIF, the initial precipitation immediately leads to a strong blockage of light with increased absorbance. Subsequently, the precipitate was gradually agglomerated into larger particles and settled down. Then the light could be able to pass through the cross section of the well again, which resulting in decreased absorbance. For the formulations prepared with 15% (F3) and 25% (F4) (*w*/*w*) TPGS, almost no turbidity was detected during the whole time period (Figure 3B). In the case of F2 prepared with 5% (*w*/*w*) TPGS, the turbidity appeared at 34 min which was approximately equal to that of F1. In contrast, the formulations prepared with SDS (F5–F7) and poloxamer 188 (F8–F10) accelerated the precipitation of ITZ (Figure 3C,D), especially for the SDS-based formulation.

### 3.4. Characterization of Optimized Formulation

The ASD formulation composed of ITZ: HPMCAS: TPGS (20:65:15/25) showed the highest drug solubility and excellent ability to maintain the supersaturated state. However, the formulation with high concentration of surfactants might lead to irritation in GI tract [18]. Therefore, in order to minimize the side effects of surfactants and maximize the absorption of ITZ in GI tract, ITZ: HPMCAS: TPGS with a ratio of 20:65:15 (F3) was selected for further physicochemical characterization to evaluate the drug physical state and drug–carrier interaction.

#### 3.4.1. Scanning Electron Microscope Analysis (SEM)

**Figure 4:**
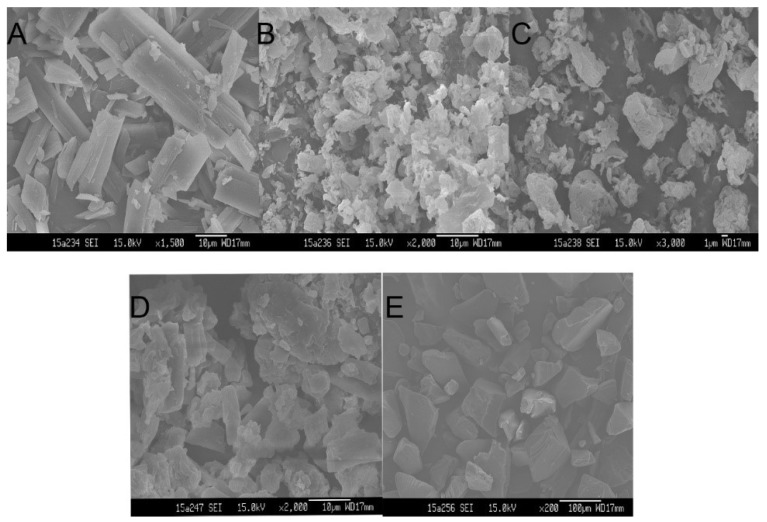
Scanning electron micrographs of (**A**) itraconazole (ITZ) powder, (**B**) Hydroxypropylmethylcellulose acetate succinate (HPMCAS), (**C**) d-α-tocopheryl polyethylene glycol 1000 succinate (TPGS), (**D**) physical mixtures, (**E**) ITZ-HPMC AS-TPGS amorphous solid dispersion.

The surface morphology of ITZ powder, HPMC AS, TPGS, physical mixtures, and the optimized ASD formulation were shown in Figure 4. ITZ power appeared as irregular prismatic-like crystalline particles with rough surface (Figure 4A). HPMC AS (Figure 4B) and TPGS (Figure 4C) appeared as amorphous particles with smooth surface. The ITZ crystals were observed to adhere around the surface of the carriers in physical mixture (Figure 4D). In contrast, no ITZ crystals were found in the optimized ASD formulation (Figure 4E), suggesting that ITZ was transformed into amorphous state. 

#### 3.4.2. Differential Scanning Calorimeter (DSC)

**Figure 5:**
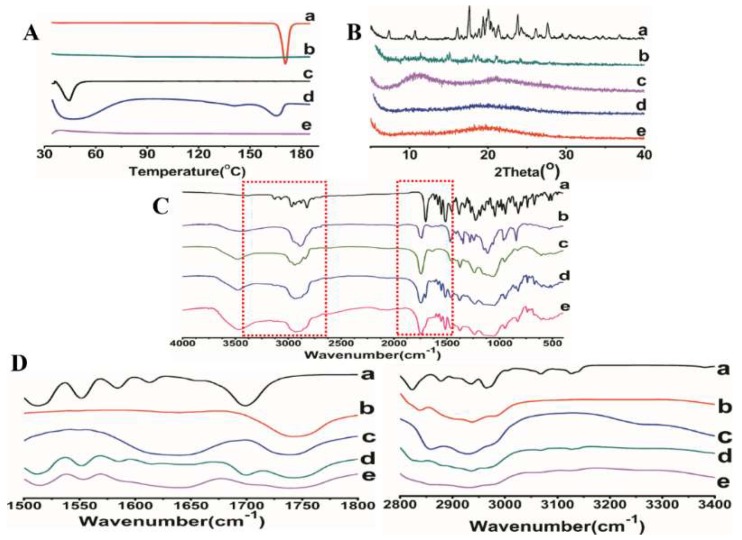
DSC (**A**), XRD (**B**), and FTIR spectra (**C**,**D**) of (**a**) ITZ powder, (**b**) HPMC AS, (**c**) TPGS, (**d**) the physical mixture of ITZ-HPMCAS-TPGS, (**e**) ITZ-HPMC AS-TPGS ASD formulation.

The DSC thermograms of ITZ, HPMC AS, TPGS, physical mixture and optimized ASD were shown in Figure 5A. The DSC curves of pure ITZ powder and TPGS showed sharp endothermic peaks at 170 °C and 39.5 °C, respectively, corresponding to their intrinsic melting points. The physical mixture showed endothermic peaks with lower intensity compared to that of pure ITZ and TPGS, indicating the good miscibility between the ITZ and the carriers. No endothermic peak was detected in the optimized ASD formulation, indicating that ITZ was converted from crystalline to amorphous state.

#### 3.4.3. Powder X-ray Diffraction (XRD)

In order to further investigate the physical state of the drug in the ASD, all the samples were investigated using powder X-ray diffraction. As shown in Figure 5B, the diffractogram of ITZ revealed its crystalline nature was detected with several characteristic diffraction peaks at 2θ equal to 11.75°, 17.80°, 18.94°, 21.84°, and 27.84°. On the contrary, the distinctive peaks of ITZ were completely disappeared in the ASD. The results were in consistent with the DSC thermograms, confirming the successful transition of crystalline ITZ into amorphous form.

#### 3.4.4. Fourier Transform Infrared Spectroscopy (FTIR)

FTIR analysis was commonly used to examine the interaction between the functional groups. Figure 5C showed the FTIR spectra of the ITZ powder, HPMCAS, TPGS, physical mixture and optimized ASD formulation. The characteristic peaks of pure ITZ were recorded at 3382, 3127, 3068, 2965, 2823, 1699, 1513, and 1451 cm^−1^. The absorption bands between 2800 and 3400 cm^−1^ was ascribed to the aromatic CH and NH_2_ groups. The peaks observed at 1613 and 1425 cm^−1^ were assigned to the C=N and C–N bonds, respectively. The sharp peaks at 1699 and 1228 cm^−1^ were observed due to the stretching of C=O and C–O, respectively (Figure 5C(a)). The FTIR spectra of physical mixture is a simple summation of those obtained from pure excipients, revealed that no interaction occurred in the ternary mixture (Figure 5D(d)). In contrast, the spectrum of ASD exhibited obvious changes in terms of the intensity and position of the absorption band (Figure 5D(e)). Compared with the pure ITZ and physical mixture, the sharp C=O peaks at 1699 cm^−1^ was disappeared and a weak stretching vibration between 2800 and 3400 cm^−1^ was observed. All the above changes confirmed the formation of hydrogen bonds between ITZ and carriers in the HPMCAS-TPGS based ASD. Hydrogen bonding is a determinant factor affecting the miscibility and physical stability of the ASDs. Strong hydrogen bonding is favorable to delay the nucleation of crystals by increasing the nucleation activation energy.

### 3.5. In Vivo Pharmacokinetic Study in Beagle Dogs

ITZ is known to be absorbed in the small intestine. Thus, in order to improve the oral bioavailability of ITZ, we prepared the ASD with enteric polymer HPMC AS incorporated with surfactant to generate a stable supersaturated solution in small intestine. Subsequently, the optimization formulation (F3) was investigated and compared with the formulation lack of TPGS (F1) and commercial product Sporanox^®^ capsules in beagle dogs.

**Figure 6:**
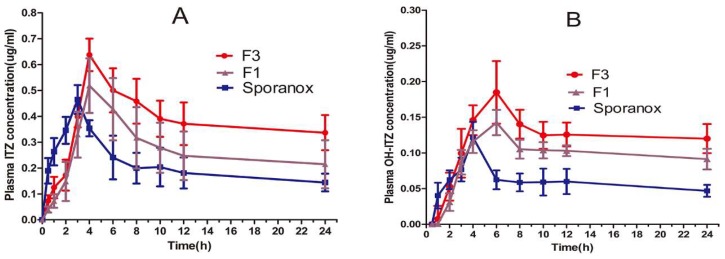
Plasma concentration–time profiles of (**A**) ITZ and (**B**) OH-ITZ after oral administration of the commercial capsules and the formulation of F1 (ITZ-HPMCAS) and F3 (ITZ-HPMCAS-15%TPGS). (Mean ± S.D., *n* = 6).

Figure 6 showed the plasma concentration-time curves of ITZ and its primary active metabolite, OH-ITZ, in beagle dogs. The pharmacokinetic parameters of ITZ and OH-ITZ were calculated according to the method mentioned in Section 2.7.2 and the results were listed in Table 3. The polymer–surfactant system (F3) resulted in significantly higher bioavailability than the commercial Sporanox^®^ and polymer system without surfactant (F1). In particular, the AUC_(0–24h) ITZ_ value of polymer–surfactant system were recorded 1.8- and 1.3-fold greater than that of commercial product Sporanox^®^ and F1, respectively. The corresponding AUC_(0–24h) OH-ITZ_ values were recorded 1.4- and 1.2-fold greater than that of Sporanox^®^ and F1, respectively. T_max_ of Sporanox^®^ was 1 h earlier than that of F1 and F3 since the HPMCAS was insoluble in stomach, resulting in delayed release of ITZ before reaching the small intestine.

## 4. Discussion

As a poorly water-insoluble drug with weak basic nature, ITZ contained ASD could rapidly solubilized in gastric fluid, which is likely to precipitate after transferring from the stomach into the small intestine, leading to a low oral bioavailability [24]. As shown in Figure 2A, the commercial product, Sporanox^®^ containing ASD prepared by HPMC achieved the highest dissolution rate in SGF. However, the dissolved ITZ tended to precipitate immediately due to the instantaneous decreased solubility in SIF. Moreover, Williams et al. proved that ITZ was primarily absorbed in the intestine because of the greater mucosal surface area for drug permeation [25,26]. Hence, the authors proposed that supersaturated solution should be targeted to intestinal for better absorption. In designing the supersaturated formulations, precipitation inhibitor is a prerequisite to maintain the poorly water-soluble drug in supersaturated sate. For these purposes, in the current work, enteric polymer HPMC AS was combined with various surfactants to screen the optimum formulation that could effectively retard ITZ precipitation.

In vitro dissolution test revealed that only 4% of ITZ was released to SGF when the ASD formulated with HPMC AS alone (F1) (Figure 2A) due to the insolubility of HPMC AS in acidic condition. After the pH switching to 6.8, the highest supersaturation level was reached within 30 min since the ITZ was rapidly released from ASD particles. However, the HPMC AS could not maintain the supersaturated state and the released ITZ precipitated gradually within an hour, which was contrary to the conclusion that HPMCAS was the effective inhibitor by absorbing onto the crystal surface [27,28]. In this study, it worth mentioning that the amorphous suspended particles are thermodynamically unstable due to the higher specific surface area and surface free energy, resulting in the undissolved ITZ aggregation (nucleation) and formation of large particles (crystal growth). The dissolution rate of ITZ increased gradually after switching the dissolution medium to SIF. However, the growth of the already formed crystal still continues, further accelerating the ITZ precipitation. Hence, in order to benefit from this formulation, precipitation must be prevented for a sufficient time period.

It has been previously demonstrated that the surfactants can affect the nucleation of crystallizing drug. In this work, relatively small amounts of surfactants (5%, 15%, 25%) were incorporated into the HPMC AS-based ASD formulations (F2–F10) to evaluate the influence of surfactant on precipitation. The ability of maintaining the supersaturation followed the order of TPGS > no surfactant ≈ poloxamer 188 > SDS, which was consistent with the result of nucleation induction time that shown in Figure 3. According to the classical nucleation theory, it is generally accepted that nucleation induction time is inversely proportional to the nucleation rate (*J*), which is expressed by the equation

(2) $$J=Aexp\left\{-\frac{16\pi v^{2}\gamma^{3}}{3k^{3}T^{3}{\left(lnS\right)}^{2}}\right\}$$

where *v* is the molecular volume of the crystallizing solute, *A* is the pre-exponential kinetic factor, *T* is the absolute temperature, *S* is the degree of supersaturation (depicted in Equation (1)), *k* is the Boltzmann’s constant, and *γ* is the interfacial energy per unit area. Equation (2) indicates that the precipitation is strongly dependent on (1) the degree of supersaturation *S* and (2) the interfacial tension *γ*.

In supersaturated solution, when the concentration of surfactants exceeding their critical micelle concentration (CMC), an improvement in drug solubility will inhibit the precipitation by reducing the degree of supersaturation (thermodynamic precipitation inhibition). In this study, the final concentrations of the surfactants in dissolution medium were in the range of 0.011–0.056 mg/mL. As the results shown in Figure 2B and Figure 3B, the ASD containing 15% and 25% TPGS significantly inhibited the ITZ precipitation since the TPGS concentration in dissolution medium was above its CMC value (0.02 mg/mL) [29]. Moreover, TPGS might also be adsorbed onto the surface of small particles thereby inhibited nucleation and crystal growth through blocking active surface and providing steric hindrance (kinetic precipitation inhibition). In addition, hydrogen bonding between ITZ and the TPGS/HPMC AS was also in the favor of maintaining the supersaturated state (Figure 5C). Structurally, the hydroxyl groups of HPMC AS and TPGS acted as hydrogen bond donors and acceptors for the ITZ. Therefore, combining TPGS and HPMC AS could exhibit a synergistic effect on inhibiting the ITZ precipitation. In the case of poloxamer, which is tri-block copolymer with polyethylene oxide (PEO)–polypropylene oxide (PPO)–polyethylene oxide (PEO) structure [30], the CMC of which was strongly dependent upon the number of polypropylene oxide (PPO) units [31]. However, poloxamer 188 used in this study has fewer PPO units result in a higher CMC value (64.67 mg/mL) [30], which was much higher than the final concentration of poloxamer 188 in the solution. Therefore, poloxamer 188 has no effect on the precipitation inhibition but could maintain the supersaturated state due to its ability to increase the wetting properties of the HPMC AS. However, the formulation containing SDS accelerated the nucleation and crystal growth (Figure 2C and Figure 3C), which could be explained by the following reasons: (1) SDS could greatly enhance the solubility of HPMC AS in SGF, thereby leading to a higher supersaturation level in SGF [19]. (2) SDS could form complex chain with polymer in solution, which could accelerate the precipitation of poorly water-soluble drug [21].

It is well known that the oral bioavailability of poorly water-soluble drugs is often limited by their insufficient dissolution. In an ASD system, the drug is dispersed at an amorphous state, and a supersaturated solution is generally formed after rapid dissolution. Therefore, ideal formulations should be able to maintain the supersaturated state to allow better absorption. In this study, three types of formulations including commercialized product Sporanox^®^, ASD prepared without surfactant (F1), and optimized formulation (F3), were administered orally to beagle dogs and their pharmacokinetic parameters were compared (Table 3). The addition of TPGS in ASD significantly improved the bioavailability as compared to F1 and Sporanox^®^ (Figure 6), which was in consistent with the results obtained from in vitro dissolution test. After comparison of the in vitro and in vivo data, it appears that there is a relatively good correlation (R^2^ = 0.91). Moreover, TPGS has been reported to be an effective oral absorption enhancer of the poorly water-soluble drugs through micelle formulation and inhibiting the biological activity of P-gp [32]. Prasad et al. also demonstrated that surfactants such as TPGS could alter the epithelium transport of drugs by changing the biophysical characteristics of the intestinal epithelial membrane [33].Taken together, the remarkable enhancement in the bioavailability of ITZ and OH-ITZ in the F3 (the optimized formulation from our in vitro screening) could be attributed to a combination of the following factors: (1) amorphous conversion of ITZ that increased the dissolution rate, (2) HPMC AS retarded the generation of supersaturation to avoid the precipitating in the stomach, (3) the synergism of TPGS-HPMC AS to inhibit the precipitation in the gastrointestinal environment, (4) reduced elimination of ITZ due to the inhibitory effect of TPGS on intestinal P-gp drug efflux.

## 5. Conclusions

This study focused on the polymer–surfactant system based on ASD to enhance the solubility of poorly water-soluble drugs and prevent the drug precipitation in supersaturated solution. The results indicated that the combination of TPGS with HPMC AS resulted in a synergistic effect to inhibit the ITZ precipitation even more significantly. In vivo study has demonstrated that the approach of generating a supersaturation in small intestinal instead of stomach is a valuable tool to enhance the absorption of poorly soluble weak bases such as ITZ.

## Figures and Tables

**Table 1:** Formulation compositions of ITZ-loaded ASDs (wt %).

	F1	F2	F3	F4	F5	F6	F7	F8	F9	F10
ITZ	20	20	20	20	20	20	20	20	20	20
HPMC AS	80	75	65	55	75	65	55	75	65	55
TPGS	-	5	15	25	-	-	-	-	-	-
SDS	-	-	-	-	5	15	25	-	-	-
Poloxamer 188	-	-	-	-	-	-	-	5	15	25

ITZ: itraconazole; HPMC AS: hydroxypropyl methylcellulose acetate succinate; TPGS: d-α-tocopheryl polyethylene glycol 1000 succinate; SDS: sodium dodecyl sulfate.

**Table 2:** Equilibrium solubility of ITZ in SIF containing different surfactants for 48 h (Mean ± S.D., *n* = 3).

Surfactants	ITZ Concentration in SIF (μg/mL)
Blank group	0.008 ± 0.002
TPGS	4.83 ± 0.81
SDS	1.66 ± 0.53
Poloxamer 188	0.92 ± 0.27
Soluplus	0.10 ± 0.06
Sodium cholate	0.07 ± 0.02
Tween 80	0.03 ± 0.01

**Table 3:** Pharmacokinetic parameters of ITZ and OH-ITZ after oral administration of the optimized formulation (F3), formulation of ITZ-HPMCAS (F1), and marketed product (Sporanox^®^) at a dose of 100 mg in beagle dogs (Mean ± S.D., *n* = 6).

Pharmacokinetic Parameter	F3	F1	Sporanox^®^
C_max_ (μg/mL), ITZ	0.61 ± 0.08 *	0.51 ± 0.12	0.45 ± 0.06
C_max_ (μg/mL), OH-ITZ	0.18 ± 0.07	0.14 ± 0.03	0.12 ± 0.04
T_max_ (h), ITZ	3.89 ± 0.41 *	4.13 ± 0.57	2.92 ± 0.37
T_max_ (h), OH-ITZ	5.64 ± 0.97	6.40 ± 0.59 *	4.60 ± 0.63
AUC_0–24h_ (μg h/mL), ITZ	8.98 ± 1.76 **	6.40 ± 1.12 *	5.06 ± 1.43
AUC_0–24h_ (μg h/mL), OH-ITZ	2.88 ± 0.76 *	2.27± 0.94 *	1.41± 0.37

* Significantly different from Sporanox^®^ according to the ANOVA test (*p* < 0.05); ** Significantly different from Sporanox^®^ according to the ANOVA test (*p* < 0.01).
